# Experimental Investigation into Stability, Heat Transfer, and Flow Characteristics of TiO_2_-SiO_2_ Hybrid Nanofluids Under Multiple Influencing Factors

**DOI:** 10.3390/nano16060359

**Published:** 2026-03-15

**Authors:** Jiahao Wu, Zhuang Li, Weiwei Jian, Danzhu Ma

**Affiliations:** 1 College of Mechanical Engineering, Liaoning Petrochemical University, Fushun 113001, China; wujiahao980410@163.com; 2 College of Petroleum Engineering, Liaoning Petrochemical University, Fushun 113001, China; jianweiwei88@lnpu.edu.cn

**Keywords:** TiO_2_-SiO_2_ nanofluids, force convective heat transfer, pressure drop measurements, heat transfer enhancement, hybrid nanofluid, minichannel heat sink

## Abstract

Extensive research and empirical evidence demonstrate that nanofluids enhance heat transfer efficiency in microchannels, but this improvement is often accompanied by increased pressure drop and particle clogging. This study aims to determine the optimal parameters for preparing stable nanofluids and to discuss the effects of different parameters on thermal and hydraulic performance. By analyzing the impact of varying ultrasonication time, particle concentration, particle size, surfactant type, and mixing ratios on stability, the most stable nanofluid was selected for evaluation of flow heat transfer and cost-effectiveness. Results indicate that a 1:1 mixed nanofluid of TiO_2_ (20 nm)-SiO_2_ (50 nm) exhibits optimal stability under conditions of 90 min ultrasonication, 0.20 vol% total particle concentration, and 0.15 wt% xanthan gum. At a Reynolds number of 550, this mixed nanofluid exhibits superior thermal performance. Compared with deionized water, its convective heat transfer coefficient and Nusselt number increase by 40.25% and 37.94%, respectively, while the pressure drop rises by only 17.18%. The performance evaluation criterion reaches 1.43, accompanied by a high cost–performance factor. These findings demonstrate that mixing large and small particles of TiO_2_ and SiO_2_ not only significantly enhances thermal performance but also positively impacts stability and hydraulic properties. A 90 min ultrasonic treatment time markedly improves stability and optimizes dynamic light scattering results.

## 1. Introduction

In recent years, the contradiction between ever-increasing energy demands and the scarcity of traditional non-renewable energy sources has intensified [1,2]. Alongside research into green sustainable energies such as solar power [3], geothermal energy [4], and hydrogen energy [5], enhancing energy utilization and thermal conversion efficiency has become paramount for contemporary societal development. Conventional heat transfer fluids, constrained by their inherent properties, often exhibit suboptimal efficiency, representing a primary form of energy loss [6]. Nanofluids [7], colloidal suspensions of nanomaterials (NMs) dispersed within base fluids such as water [8], oil [9], or ethylene glycol (EG) [10], can significantly enhance the thermal properties of the base fluid, thereby improving heat transfer efficiency. The role of nanofluids within nanotechnology is paramount. Benefiting from the diverse range of nanoparticle materials available, nanofluids exhibit varied properties suitable for various applications, holding promise to revolutionize fields such as electrical engineering [11,12], the automotive industry [13,14,15], aerospace [16], and renewable energy applications, including solar power [17,18,19,20,21,22]. The thermophysical properties (TPPs) of nanofluids, including thermal conductivity (TC), convective heat transfer coefficient, and dynamic viscosity (DV), are paramount [23,24]. These characteristics drive the effectiveness and enhanced performance of heat transfer systems [25,26].

To date, nanoparticles are commonly categorized into three main classes: metallic, non-metallic, and hybrid [27,28]. Metallic nanoparticles can be further subdivided into pure metals [29], metal oxides [30,31,32,33], and others. Non-metallic materials chiefly encompass carbon-based nanoparticles [34], ceramic particles [35], and phase change materials (PCMs) [36]. Among metallic materials, pure metal nanoparticles—particularly gold and silver—exhibit remarkable thermal conductivity and localized surface plasmon resonance (LSPR) effects [37], conferring significant advantages for thermal energy conversion. However, they are also among the most costly materials [38]. In contrast, metal-oxide nanofluids (NFs) such as TiO_2_ [39,40], ZnO [13,41], Al_2_O_3_ [42], and Fe_3_O_4_ [43] are widely used due to their cost-effectiveness, ease of synthesis, and superior thermal conductivity. Ceramic nanoparticles are attracting increasing interest due to their extensive engineering applications. Among these, SiO_2_ stands out as a highly promising nanomaterial for heat transfer owing to its outstanding thermophysical properties, including exceptional thermal stability, mechanical strength, and rigidity [44,45]. Both TiO_2_ and SiO_2_ nanoparticles are also commonly employed as nanolubricants [46,47].

Tuckerman and Pease introduced the concept of microchannels (MCHS) [48]. Due to their smaller geometry and low coolant requirements, they enhance the compactness and performance of cooling devices [49,50,51,52,53]. However, this advantage also leads to potential issues when MCHS are combined with nanofluids—particularly those with poor stability—as deposition can readily occur. As numerous particles collide with the walls and clog the channels, the nanofluid loses its high-efficiency characteristics. Furthermore, fouling degrades MCHS, thereby increasing energy consumption and shortening device lifespan [54,55,56].

Despite the significant potential for nanofluid applications, they face key challenges, including insufficient nanoparticle stability, susceptibility to aggregation, and poor dispersibility. These issues constrain their thermophysical properties and long-term performance [57,58]. Therefore, achieving enhanced stability alongside optimized thermophysical properties has emerged as a paramount challenge and central research thrust in nanofluid science. The stability of nanofluids can be analyzed by examining the forces acting on suspended nanoparticles. Within a nanofluid system, the primary particle–particle and particle–fluid interactions include van der Waals forces [59], electrostatic forces [60,61], gravity [62], and Brownian forces, which arise from [63] the random thermal motion of nanoparticles. Among these, the electric double-layer force [64] is particularly significant. This force represents a specific manifestation of electrostatic interactions within liquid media, playing a pivotal role in numerous colloidal and biological systems. It directly influences stability and rheological properties and, together with van der Waals forces, forms the foundation of DLVO theory [65]—a cornerstone of colloid science.

The stabilization mechanisms of nanofluids encompass electrostatic stabilization [66,67], steric stabilization [68], and hydrogen bonding stabilization [69]. The diverse chemical and physical properties that result from combining different nanoparticle additives significantly broaden the application potential. Numerous studies indicate that hybrid nanofluids exhibit enhanced thermal conductivity and often improved stability compared to single-component systems [70,71]. The following methods are commonly employed for characterizing the stability of nanofluids: (1) Sedimentation observation [72]: Stability is assessed by examining photographs of nanofluid sedimentation over time; quantitative analysis is achievable through sedimentation coefficient calculation using added scale bars. (2) Zeta potential analysis [73,74]: Reflects the repulsive force between nanoparticles; higher zeta potential values indicate stronger interparticle repulsion. (3) Absorbance measurement [38,75]: Based on the Lambert–Beer law, this method captures concentration changes during nanofluid sedimentation by measuring the absorbance of the upper solution layer. (4) Particle size analysis [76,77]: This method assesses agglomeration by analyzing the particle size distribution in the solution.

Although previous research on nanofluids has been extensive, and studies on nanofluids within MCHS have been diverse, investigations into the stability of working fluids have been limited, primarily focusing on simulating the sedimentation rates of nanoparticles in MCHS applications. While numerous studies have demonstrated that mixed nanofluids significantly enhance thermal performance, this improvement is often accompanied by increased pressure drop. Furthermore, due to the influence of nanofluid stability, particle sedimentation and clogging can occur easily, affecting the lifespan of MCHS. Although consensus has emerged in recent years regarding enhanced heat transfer from mixing large and small particles, no research has yet addressed their potential to increase stability and reduce pressure-drop friction. This paper first examines multiple factors affecting nanofluid stability through characterization analysis and mechanism investigation. The most stable nanofluid is then selected for flow heat transfer experiments to validate its thermal properties and thermal economics. This study identifies the preparation parameters for stable nanofluids and discovers and clarifies the impact of particle-size mixing in hybrid nanofluids on enhancing stability and hydraulic performance.

## 2. Materials and Methods

### 2.1. Materials

#### 2.1.1. Nanoparticles

The specifications of the nanoparticles used in this study are shown in Table 1. Both samples were purchased from Shanghai Macklin Biochemical Technology Co., Ltd. (Shanghai, China), with a purity of 99.5%.

#### 2.1.2. Surfactant

Nanoparticle agglomeration compromises the performance of nanomaterials by degrading key properties such as thermal conductivity and colloidal stability. The addition of surfactants mitigates this issue by reducing the surface tension of the base fluid, thereby preventing particle aggregation and extending nanofluid stability. Given the variety of surfactant-stabilization mechanisms, surfactant type and concentration must be carefully optimized when formulating stable nanofluids. In this study, surfactants with different ionic characteristics were systematically analyzed. The surfactants employed are listed in Table 2. CMC, CTAB, PVP, and XG powders were procured from Shanghai Macklin Biochemical Technology Co., Ltd., while TA powder was procured from Tianjin Kermel Chemical Reagents Co., Ltd. (Tianjin, China).

### 2.2. Methods and Characterization

#### 2.2.1. Preparation and Experimental Procedure of Nanofluids

**Figure 1:**
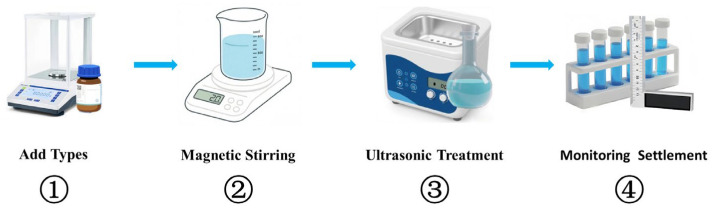
Preparation process of nanofluids.

To enable consistent, comparable sedimentation analysis, nanoparticle concentration is expressed as a volume concentration (vol%). As illustrated in Figure 1, a two-step method was employed to prepare nanofluids. First, the required mass of nanoparticles corresponding to the target volume concentration was calculated using Equation (1) and accurately weighed using a high-precision analytical balance (HC1002X, Hochoice, Shanghai, China). The weighed particles were then dispersed in an appropriate amount of deionized water. Subsequently, the suspension was stirred by a magnetic stirrer (AS-DF-101S, Ancssy, Shanghai, China) at 600 rpm for 60 min to achieve initial wetting and preliminary dispersion. Finally, to further break down agglomerates and enhance dispersion stability, the mixture was treated in an ultrasonic bath (AK-040SD, Yuclean, Shenzhen, China) for 30–120 min. The ultrasonic bath water was replaced every half hour to prevent overheating of the nanofluid.

(1) $$\emptyset =\left(\begin{array}{c} \frac{m_{p}}{\rho_{p}}\\ \frac{m_{p}}{\rho_{p}}+\frac{m_{bf}}{\rho_{bf}} \end{array}\right)$$

where $m_{p}$ is the mass of nanoparticles, $m_{bf}$ is the mass of the base fluid, $\rho_{p}$ is the density of nanoparticles, $\rho_{bf}$ is the density of the base fluid, and ∅ is the volume fraction of nanoparticles. The volume concentration equals the volume fraction multiplied by 100%.

**Figure 2:**
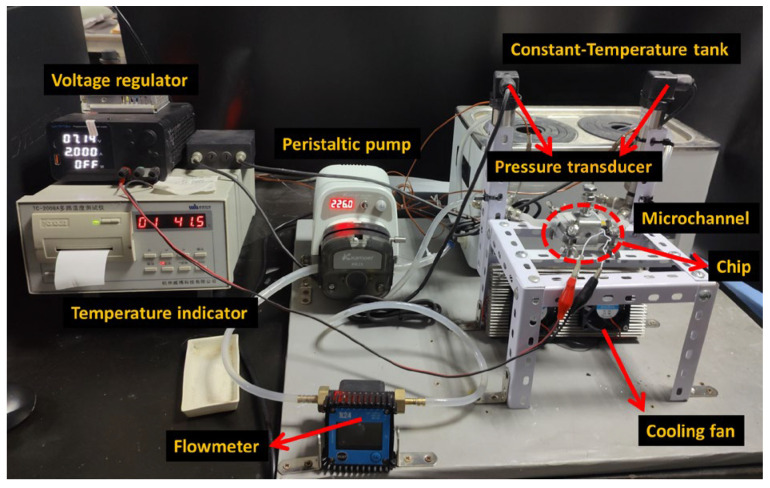
Photograph of the experimental setup for flow and heat transfer measurements.

**Figure 3:**
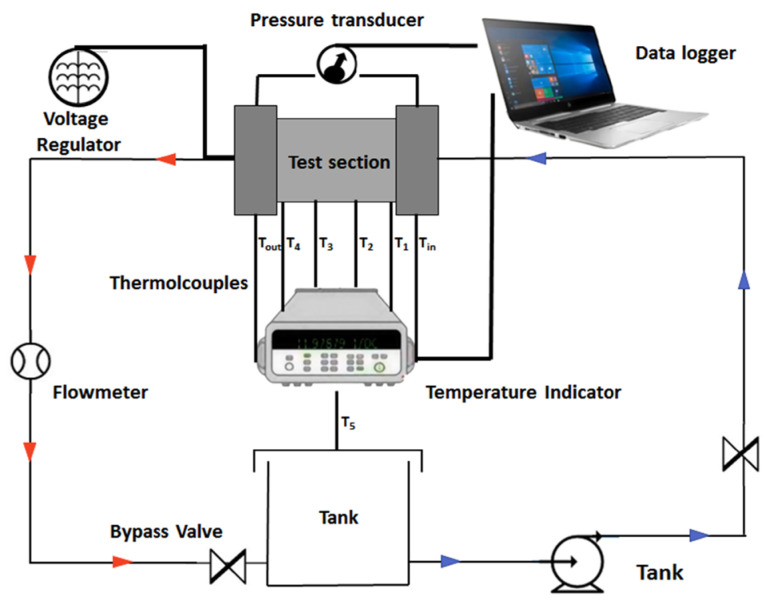
Schematic of the experimental setup for flow and heat transfer measurements.

Heat exchange experiments were conducted using a custom-built platform, as illustrated in Figure 2. The experimental setup comprised a temperature indicator (TC-2008A, Hzwb, Hangzhou, China), a pressure transducer (SUP-P300, Lontrol, Hangzhou, China), a flow meter (K24, Kavit, Shanghai, China), type K thermocouples (KPS-QB-K-2, Kaipusen, Xinghua, China), a reservoir tank (WB100-2, Joanlab, Huzhou, China), a peristaltic pump (DIP1500-S183-GB, Kamoer, Shanghai, China), flexible tubing, a voltage regulator (TPS605, Wanptek, Shenzhen, China), a test chip (XH-RP4040, Tsl, Suqian, China) with an integrated MCHS, and a data acquisition system connected to a computer for real-time logging. The schematic diagram of the apparatus is shown in Figure 3.

The MCHS was 3D-printed using aluminum alloy with a thermal conductivity of 120 W/m·K to ensure precision and appropriate dimensions. The straight MCHS consists of four parallel rectangular microchannels, each measuring 40 mm in length, 1.5 mm in width, 2 mm in depth, and spaced 2 mm apart. The surface roughness of the MCHS was in the range of Ra 12.5–25 μm.

#### 2.2.2. Characterization Methods

The stability and dispersion characteristics of the nanofluids were assessed through multiple analytical techniques. The absorption spectra (Abs) of the nanofluids were measured using a UV-Vis-NIR spectrophotometer (Cary Series, Agilent Technologies, Santa Clara, CA, USA). The zeta potential was determined to evaluate the electrostatic stability of the suspensions. Furthermore, the particle size distribution within the nanofluids was analyzed via dynamic light scattering (DLS) using a laser particle size analyzer (Nano-ZS90, Malvern Instruments, Malvern, UK).

#### 2.2.3. Analysis of Nanofluid Stability

**Figure 4:**
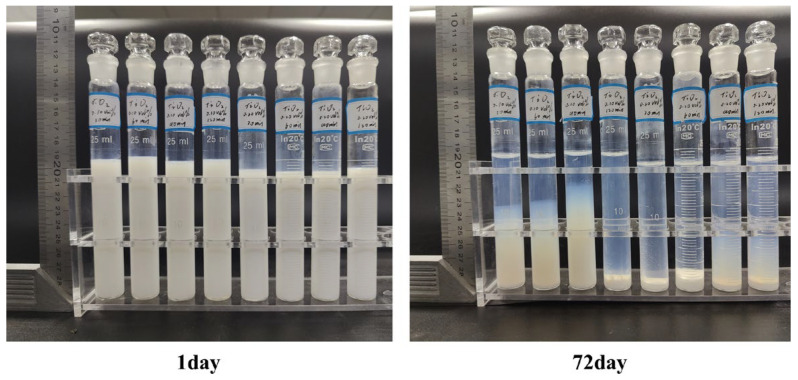
Sedimentation coefficient observation method for TiO_2_ nanofluids under identical surfactant conditions (0.15 wt% CMC) at different concentrations (0.10 vol% and 0.20 vol%) and varying ultrasonication durations (30–120 min).

The stability of nanofluids is commonly evaluated qualitatively by imaging the suspensions and recording the visual changes in sedimentation over time. This study employs a novel sedimentation observation technique. As shown in Figure 4, during the initial preparation stage, both concentrations (0.10 vol% and 0.20 vol%) of the nanofluid (TiO_2_, 0.15 wt% CMC) exhibited excellent stability and uniformity across different ultrasonication durations (30–120 min). Even after 72 days of preparation, some nanofluids maintained high stability, with the 0.10 vol% TiO_2_ nanofluid treated for 90 min demonstrating particularly outstanding performance. Simultaneously, it is evident that for high-concentration TiO_2_ nanofluids, extended ultrasonication durations, such as 90 and 120 min, significantly enhance stability; however, this cannot fully compensate for their substantial concentration disadvantage. In this approach, the nanofluid sample vial and a calibrated scale were positioned together within a fixed field of view for daily imaging. The sedimentation front in each image was then measured multiple times using Nano Measurer 1.2 software to minimize observational error. Each set of measurements was repeated six times to reduce random variation. Finally, the sedimentation coefficient was calculated according to Equation (2), providing a quantitative assessment of nanofluid stability. By adopting this method, comparisons based on volume concentration offer a more objective and equitable basis for stability evaluation than those based on mass concentration.

(2) $$K=\frac{L_{0}}{L}$$

In this context, *K* represents the sedimentation coefficient (or sedimentation rate) of the nanofluid. A lower value of *K* indicates better stability of the nanofluid, whereas a higher value corresponds to greater sedimentation. Here, *L*_0_ denotes the height (in mm) of the clear supernatant layer after the nanofluid has undergone static sedimentation, and *L* represents the total height (in mm) of the initial nanofluid suspension.

Optical spectroscopy is widely employed to evaluate the stability of nanofluids. This method is based on the Beer–Lambert law, which states that absorbance is linearly proportional to the concentration of nanoparticles in suspension [78,79]. The stability of a nanofluid is determined by measuring its absorbance and monitoring changes over time; a higher and more stable absorbance value generally indicates better colloidal stability.

However, this approach is not directly applicable to dark or highly concentrated nanofluids, as intense light absorption or scattering can interfere with optical measurements. For instance, Wang et al. [80] diluted black graphene-based nanofluids at a 1:50 ratio prior to absorbance measurements. In the present study, to ensure reliable detection within the instrument’s measurable range (up to an absorbance of 10), the supernatant of the prepared nanofluid was extracted and diluted at multiple ratios: 1:10, 1:20, 1:30, 1:40, and 1:50. Experimental tests revealed that for a 0.2 vol% milky TiO_2_-SiO_2_ nanofluid, a dilution ratio of 1:30 provided the optimal balance between detection accuracy and signal linearity.

#### 2.2.4. Thermal–Physical Properties of Nanofluids and Data Processing

The dynamic viscosity, thermal conductivity, density, and specific heat capacity represent fundamental thermophysical properties of nanofluids. This study draws upon the work of Asif Khan et al. [81] on Al_2_O_3_-SiO_2_ nanofluids in a straight minichannel heat sink, employing the Brinkman model [82], while the thermal conductivity of hybrid nanofluids was predicted using the Hamilton–Crosser formula [83], as given in Equations (3) and (4), respectively.

(3) $$\mu_{nf}=\frac{\mu_{bf}}{{\left(1-\varphi_{np}\right)}^{2.5}}$$

(4) $$\frac{k_{hnf}}{k_{bf}}=\left[\frac{\left(\frac{\varphi_{np1}k_{np1}+\varphi_{np2}k_{np2}}{\varphi }+(n-1)k_{bf}+(n-1)(\varphi_{np1}k_{np1}+\varphi_{np2}k_{np2})\right)-(n-1)\varphi k_{bf}}{\left(\frac{\varphi_{np1}k_{np1}+\varphi_{np2}k_{np2}}{\varphi }+(n-1)k_{bf}+\left(\varphi_{np1}k_{np1}+\varphi_{np2}k_{np2}\right)\right)-\varphi k_{bf}}\right]$$

The density and specific heat capacity of hybrid nanofluids were estimated using empirical expressions proposed by Takabi and Salehi [84], corresponding to Equations (5) and (6), respectively.

(5) $$\rho_{hnf}=(\varphi_{np1}\rho_{np1})+(\varphi_{np2}\rho_{np2})+(1-\varphi_{np1}-\varphi_{np2})\rho_{bf}$$

(6) $$C_{p,lnf}=\left(\frac{\left(\varphi_{np1}\rho_{np1}C_{p,np1}\right)+\left(\varphi_{np2}\rho_{np2}C_{p,np2}\right)+\left(1-\varphi_{np1}-\varphi_{np2}\right)\rho_{bf}C_{p,bf}}{\rho_{hnf}}\right)$$

The Reynolds number can be calculated using Equation (7):

(7) $$R_{e}=\frac{\rho \nu d_{h}}{\mu }$$

where ν is the characteristic mean flow velocity and $d_{h}$ represents the hydraulic diameter of the heat exchanger, calculated by Equations (8) and (9):

(8) $$v=\frac{\dot{V}}{{N_{ch}W}_{ch}H_{ch}}$$

(9) $$d_{h}=\frac{4A_{ch}}{p}=\frac{4W_{ch}H_{ch}}{2(W_{ch}+H_{ch})}$$

where $W_{ch}$ and $H_{ch}$ are the cross-sectional width and height of an individual channel.

The heat transfer rate Q between the heat exchanger and the fluid is calculated using Equations (10) and (11).

(10) $$Q=\dot{m}C_{p}(T_{out}-T_{in})$$

(11) $$\dot{m}=\dot{V}\rho$$

where *Q* is the heat flux, $\dot{m}$ is the mass flow rate, $C_{p}$ is the specific heat capacity, $T_{out}$ is the outlet temperature of the coolant, $T_{in}$ is the inlet temperature, and $\dot{V}$ is the volumetric flow rate.

The convective heat transfer coefficient can be calculated using Equations (12) and (15):

(12) $$h=\frac{Q}{NA_{c}\Delta T_{m}}$$

(13) $$A_{c}=W_{c}L_{c}$$

(14) $$\Delta T_{m}=T_{W}-\frac{T_{out}+T_{in}}{2}$$

(15) $$T_{W}=T_{b}-\left(\frac{QH_{w}}{k_{hs}A_{w}}\right)$$

where $A_{c}$ is the heat transfer area of a single channel, $\Delta T_{m}$ is the temperature difference between the channel wall and the working fluid, $T_{W}$ is the wall temperature, $T_{b}$ is the temperature measured by the substrate-embedded thermocouple, $H_{w}$ is the distance from the channel wall to the thermocouple (2.0 mm below the wall surface), $k_{hs}$ is the thermal conductivity of the heat-sink material (120 W/m·K), and $A_{w}$ is the surface area of the un-finned wall section, calculated from Equation (16):

(16) $$A_{w}=W_{hs}L_{hs}$$

where $W_{hs}$ and $L_{hs}$ represent the width and length of the heat-sink segment under investigation, respectively.

The heat transfer performance is determined by the Nusselt number, which can be calculated using Equation (17):

(17) $$Nu=\frac{hd_{h}}{k_{c}}$$

where $k_{c}$ is the thermal conductivity of the working fluid. The heat dissipation performance of the fluid is fundamentally governed by convective thermal resistance.

As the heat dissipation performance of a fluid is fundamentally influenced by convective thermal resistance, the convective thermal resistance of fluid flow may be calculated using Equation (18):

(18) $$R_{th}=\frac{\left(T_{w}-T_{in})(T_{w}-T_{out}\right)}{Q\cdot ln\left(\frac{T_{w}-T_{in}}{T_{w}-T_{out}}\right)}$$

The pressure drop across the channel can be calculated using Equation (19):

(19) $$\Delta P=\Delta P_{m}-(K_{C}+K_{E})\frac{\rho \nu^{2}}{2}$$

where $\Delta P_{m}$ is the measured pressure difference, and $K_{C}$ and $K_{E}$ are the contraction coefficient and expansion coefficient, respectively. The contraction coefficient and expansion coefficient can be defined by Equations (20) and (21):

(20) $$K_{E}={\left(1-\frac{A_{out}}{A_{eff}}\right)}^{2}$$

(21) $$K_{C}=0.5\left[1-{\left(\frac{A_{in}}{A_{eff}}\right)}^{2}\right]$$

where $A_{eff}$ is the effective area of the heat sink, which can be estimated by Equation (22):

(22) $$A_{eff}=N_{ch}\left(W_{ch}+2\eta_{fin}H_{ch}\right)L_{ch}$$

where $N_{ch}$, $W_{ch}$, $\eta_{fin}$, $H_{ch}$ and $L_{ch}$ denote the number of channels, channel width, fin efficiency, channel height, and channel length, respectively. The fin efficiency can be calculated using Equations (23) and (24):

(23) $$\eta_{fin}=\frac{\tanh\left(m_{fin}H_{ch}\right)}{m_{fin}H_{ch}}$$

(24) $$m_{fin}=\sqrt{\frac{2h}{k_{hs}W_{fin}}}$$

The pumping power can be calculated using Equation (25):

(25) $$P_{p}=\dot{V}\Delta P$$

The friction factor for flow through the channel can be calculated using Equation (26):

(26) $$f=\frac{2d_{h}\Delta P}{\rho \nu^{2}L_{ch}}$$

The performance evaluation criterion can be calculated using Equation (27):

(27) $$PEC=\frac{Nu_{hnf}/Nu_{bf}}{{\left(f_{hnf}/f_{bf}\right)}^{1/3}}$$

The price performance factor for nanofluids comprehensively considers heat transfer performance and economic costs. It is defined as the ratio of the performance evaluation criterion to price, representing the performance evaluation criterion benefits per unit price. It can be calculated using Equation (28) proposed by Xuan et al. [85]:

(28) $$PPF=\frac{PEC}{\sum_{n}^{i=1}price}$$

#### 2.2.5. Uncertainty Analysis

Currently, every experimental investigation requires a comprehensive uncertainty analysis. The experimental uncertainties in the directly measured parameters originate from the intrinsic accuracy limits of the sensing instruments. The pressure drop, heating power, dimensions, temperature, and volumetric flow rate were measured with the following instruments and respective accuracies: a pressure transducer (±0.25% FS), a power meter (±0.5%), a vernier caliper (±0.02 mm), type K thermocouples (±1%), and a flow meter (±0.5%). Accordingly, as shown in Equation (29), the method of Moffat [86] was used to determine the uncertainty associated with the measured parameters, accounting for the accuracy of each instrument used. Based on this equation, the experimental uncertainty was calculated to be 1.60%.

(29) $$\delta y=\sqrt{{\left(\frac{\partial y}{\partial x_{1}}\delta x_{1}\right)}^{2}+{\left(\frac{\partial y}{\partial x_{2}}\delta x_{2}\right)}^{2}+{\left(\frac{\partial y}{\partial x_{3}}\delta x_{3}\right)}^{2}+{\left(\frac{\partial y}{\partial x_{4}}\delta x_{4}\right)}^{2}+{\left(\frac{\partial y}{\partial x_{5}}\delta x_{5}\right)}^{2}}$$

## 3. Results and Discussion

### 3.1. Influence of Intrinsic Nanoparticle Parameters

#### 3.1.1. Nanoparticle Concentration

**Figure 5:**
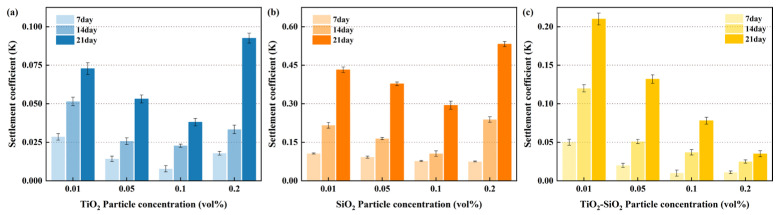
Sedimentation coefficient versus concentration for nanofluids containing 20 nm TiO_2_ (**a**), 50 nm SiO_2_ (**b**), and TiO_2_-SiO_2_ (**c**) nanoparticles under identical conditions (0.15 wt% CMC, 90 min ultrasonication time).

As shown in Figure 5, under identical experimental conditions, the sedimentation coefficients of single-component nanofluids composed of TiO_2_ and SiO_2_ within the concentration range of 0.01–0.20 vol% first decrease and then increase with rising particle concentration. Both exhibit a minimum value at 0.10 vol%, revealing a distinct numerical variation pattern. This indicates that for nanofluids, the lowest sedimentation rate—and thus the highest stability—occurs at a particle concentration of 0.10 vol%. The sedimentation coefficient of TiO_2_-SiO_2_ nanofluid decreases with increasing concentration within this range, reaching its minimum value at 0.20 vol% concentration, the point of optimal stability. This observed trend aligns well with classical colloid theory. In surfactant-free systems, at low particle concentrations, van der Waals forces—which originate from intermolecular dipole interactions and represent an attractive potential—are relatively weak. Given the nanoscale dimensions of the particles, van der Waals interactions often dominate interparticle interactions, critically influencing aggregation, dispersion stability, and self-assembly. These forces are strongly distance-dependent, increasing significantly at shorter interparticle separations, and are also affected by particle geometry, size, and surface characteristics.

At low concentrations, Brownian motion is limited, while electrostatic repulsion becomes more pronounced. However, if the Debye length (electrical double-layer thickness) exceeds the average interparticle spacing, the electrostatic repulsion may become insufficient to overcome van der Waals attraction, leading to particle agglomeration. Additionally, insufficient steric hindrance at low concentrations limits the ability to maintain colloidal stability through entropic stabilization mechanisms. As concentration increases, Brownian motion intensifies. Although enhanced particle crowding improves potential steric contributions and reduces the relative influence of electrostatic forces, the significantly amplified van der Waals attraction at high concentrations becomes the dominant driver of particle aggregation.

Therefore, an optimal concentration exists that balances these competing interactions. In this study, the optimum concentrations for TiO_2_, SiO_2_, and TiO_2_-SiO_2_ (1:1) nanofluids were determined to be 0.10 vol%, 0.10 vol%, and 0.20 vol%, respectively. The enhanced stability of the TiO_2_-SiO_2_ hybrid system at 0.20 vol% can be attributed to the favorable interfacial compatibility between TiO_2_ and SiO_2_ nanoparticles, combined with the individual stability optima of each component observed at 0.10 vol%.

#### 3.1.2. Nanoparticle Size

**Figure 6:**
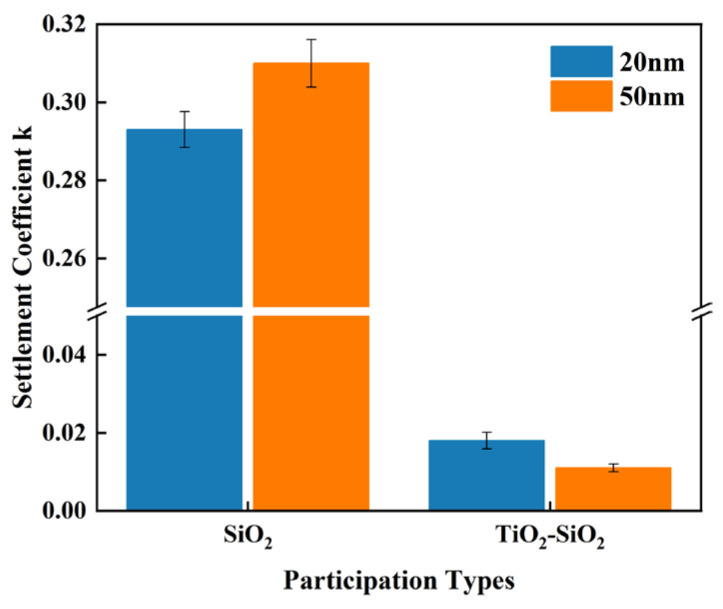
Comparison of Sedimentation Coefficients of Nanofluids Composed of SiO_2_ Nanoparticles with Different Particle Sizes under Identical Conditions (0.15 wt% CMC, 90 min Ultrasonication) after one week.

Generally, smaller particles exhibit slower sedimentation rates due to enhanced Brownian motion. As shown in Figure 6, after one week of preparation, the sedimentation coefficient of 20 nm silica particles in the single-component system was slightly lower than that of 50 nm silica particles. In contrast, the TiO_2_-SiO_2_ mixed system exhibits a different trend: over the same time period, the sedimentation coefficient of the TiO_2_-SiO_2_ mixed nanofluid containing 50 nm SiO_2_ particles is significantly lower than that of the TiO_2_-SiO_2_ mixed system containing 20 nm SiO_2_ particles, indicating reduced settling and enhanced stability. Quantitative analysis indicates that under identical conditions, the sedimentation coefficient of the TiO_2_-SiO_2_ hybrid nanofluid containing 50 nm SiO_2_ particles was only 61.11% of that in the TiO_2_-SiO_2_ hybrid nanofluid system containing 20 nm SiO_2_ particles.

This demonstrates that mixing large and small particles enhances the colloidal stability of nanofluids. The underlying heat transfer mechanism is often explained as a “particle size synergy effect”: small particles fill the gaps formed by large particles, creating a denser, continuous “large-particle–small-particle–matrix fluid” heat transfer pathway. This arrangement effectively increases the solid–liquid interface area and enhances the effective fluid density near the particle surfaces. The resulting denser, more uniform particle network not only improves heat transfer but also elevates overall heat exchange performance. Beyond verifying its enhancement of heat transfer capability, this study also employed methods to quantify nanofluid stability, concluding that mixing large and small particles significantly improves the stability of nanofluids. The mechanism by which mixed-size particle fluids influence stability, heat transfer performance, and hydraulic performance should be analogous, representing positive effects arising from the optimization of fluid properties. The underlying mechanisms may require further in-depth investigation.

#### 3.1.3. Proportioning of Nanofluid Mixture Systems

**Figure 7:**
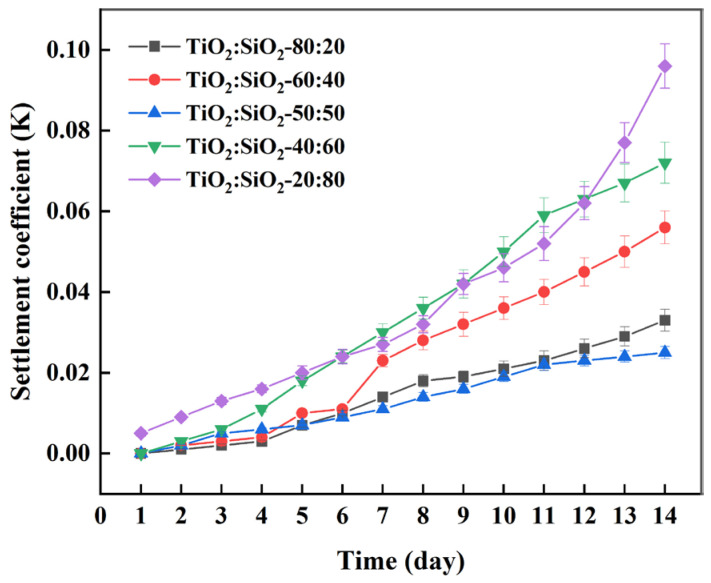
Sedimentation coefficient of TiO_2_-SiO_2_ nanofluids with different mixing ratios as a function of time under identical conditions (0.15 wt% CMC, 90 min ultrasonication).

As shown in Figure 7, under identical conditions, the sedimentation coefficient curves for all TiO_2_-SiO_2_ nanofluid blends at 0.20 vol% exhibit relatively smooth variation, which may be attributed to the favorable compatibility and intrinsic stability between TiO_2_ and SiO_2_ nanoparticles. During the first four days, higher TiO_2_ content corresponded to better stability in the hybrid system, likely due to the superior inherent stability of TiO_2_ nanoparticles. However, after the fifth day, the 50:50 blend showed the highest stability. This may be explained by the previously observed optimal stability of individual 0.10 vol% TiO_2_ and SiO_2_ nanofluids, and it may also imply that in a binary hybrid system, a higher content of the more stable component does not necessarily lead to better overall stability. Therefore, the 50:50 ratio is identified as the optimal blend for TiO_2_-SiO_2_ nanofluids in this study.

### 3.2. Impact of Methods for Enhancing Nanofluid Stability

#### 3.2.1. Ultrasonic Duration

**Figure 8:**
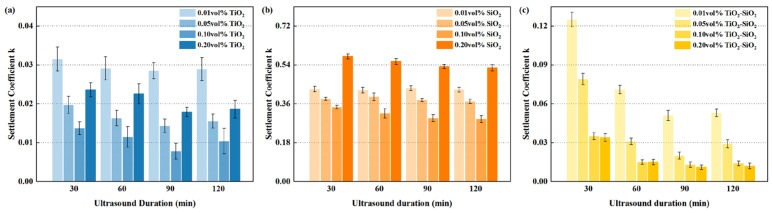
Variation in sedimentation coefficient with ultrasonication time for nanofluids containing 20 nm TiO_2_ (**a**), 50 nm SiO_2_ (**b**), and TiO_2_-SiO_2_ (**c**) nanoparticles under identical conditions (0.15 wt% CMC, 90 min ultrasonication) after one week.

As shown in Figure 8, under ultrasonication times ranging from 30 to 120 min, the sedimentation coefficients of TiO_2_ and TiO_2_-SiO_2_ nanofluids at various concentrations initially decreased and then increased with prolonged ultrasonication, reaching a minimum at 90 min. This trend is consistent with the findings reported by Mahbubul et al. [87] and Feng et al. [88]. In contrast, the minimum sedimentation coefficient for SiO_2_ nanofluids was observed at 120 min of ultrasonication. Furthermore, for all nanofluids across the four concentrations (0.01–0.20 vol%), the overall effect of different ultrasonication times remained largely consistent, indicating that the influence of ultrasonication duration on nanofluid stability is independent of concentration. Possible effects of ultrasonication duration on nanofluid stability: Prolonged ultrasonication induces solution temperature elevation, altering interactions between surfactants and nanoparticles. This facilitates tighter, more uniform adsorption of surfactant molecules onto particle surfaces, thereby enhancing nanofluid stability. However, prolonged duration and high temperatures can lead to adverse effects. For instance, Mahbubul et al. [87] systematically investigated the impact of ultrasonic duration on Al_2_O_3_ nanofluids, finding that particles re-agglomerate beyond the optimal time. Abd Malek et al. [89] observed that increased ultrasonic duration caused temperature changes in MXene nanofluids, triggering particle agglomeration. Chakraborty et al. [90] found that prolonged ultrasonication resulted in a temperature rise, leading to decreased stability in TiO_2_ nanofluids. Moreover, we can clearly observe that the TiO_2_-SiO_2_ nanofluid exhibits significant sensitivity to ultrasonic treatment duration, with particularly pronounced differences in sedimentation coefficients occurring between 30 and 90 min.

Zeta potential measurements were conducted on the prepared nanofluids. Interestingly, the results revealed a distinct deviation from the conventional correlation between zeta potential and colloidal stability. As summarized in Table 3, the TiO_2_ nanofluid with the lowest sedimentation (90 min ultrasonication) exhibited the lowest recorded zeta potential. In contrast, the least stable sample (30 min ultrasonication) displayed a significantly higher zeta potential of −53.1 mV. This suggests that while zeta potential generally serves as an indicator of electrostatic stabilization, high stability in certain systems can coexist with a low zeta potential [91].

The observed anomaly is likely due to ultrasonication-induced surface modifications. Prolonged ultrasonication is known to generate cavitation bubbles, which can create surface defects on TiO_2_ nanoparticles, typically in the form of oxygen vacancies or Ti^3+^ species [92,93]. These defects enhance surface activity [94,95] and may alter interfacial charge distribution, thereby reducing the effective electrostatic repulsion and leading to a lower measured zeta potential. This mechanism aligns with reports on ultrasonicated TiO_2_ in other applications. For instance, Bae et al. [96] demonstrated that an ultrasonic transducer–TiO_2_ assembly improved DSSC efficiency by approximately 45% compared to TiO_2_ alone, attributed to surface activation via ultrasonic treatment. This may also stem from ultrasonication causing particle fragmentation, which drastically increases the total surface area of the particles. If the total amount of charged functional groups on the particle surface (such as carboxyl or hydroxyl groups) does not increase synchronously, the surface charge density per unit area will decrease. This would directly lead to a reduction in the absolute value of the zeta potential. Additionally, prolonged ultrasonic treatment may elevate solution temperature, altering surfactant properties such as chemical bond breakage [97], thermal degradation, and cloud point [98], which may subsequently modify the zeta potential.

A comparable phenomenon has been documented for ZnO nanofluids, where ultrasonication introduced surface defects (e.g., oxygen vacancies or Zn^2+^) that enhanced surface activity and reduced zeta potential [99,100]. This behavior appears characteristic of certain metal-oxide nanofluids and may contribute to their observed stability despite low zeta potential readings. Consequently, in systems where surface activation dominates over electrostatic repulsion, stability may be maintained through mechanisms other than high zeta potential, such as enhanced steric or electrosteric effects arising from defect-mediated surface modification.

**Figure 9:**
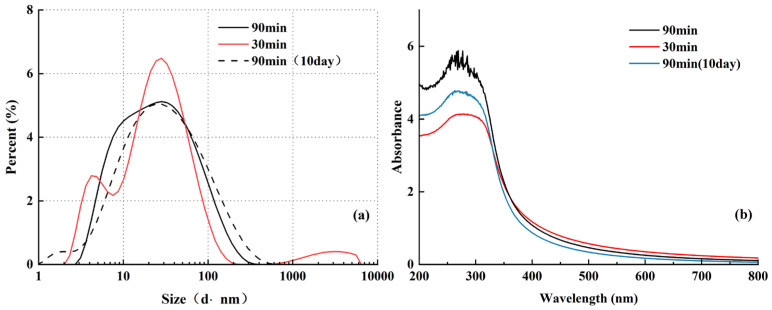
Effect of ultrasonication time on 0.20 vol% TiO_2_-SiO_2_ (0.15 wt% XG) nanofluids: (**a**) hydrodynamic diameter distribution measured by dynamic light scattering (DLS); (**b**) UV–Vis absorbance spectra.

The DLS results presented in Figure 9a show that the majority of particles fall within a narrow size range of approximately 20–50 nm, indicating good dispersion and consistent hydrodynamic diameter distribution. In the sample subjected to 30 min of ultrasonication, the highest proportion of 20 nm particles was observed, along with the presence of large agglomerates. This suggests that shorter ultrasonication times may result in insufficient particle de-agglomeration or rapid re-agglomeration. In contrast, the sample treated for 90 min exhibited a more uniform hydrodynamic diameter distribution, with minimal agglomeration, further supporting the influence of ultrasonication duration on nanofluid stability and corroborating the sedimentation coefficient (K) measurements. After 10 days, the DLS curve shifted slightly to the right, without the appearance of new peaks, indicating that ultrasonication effectively disrupted nanoparticle agglomerates and that the dispersion remained stable, without forming large aggregates over time.

The absorption (Abs) spectra in Figure 9b reveal that on the first day after preparation, the sample ultrasonicated for 90 min exhibited higher absorbance, with a peak value of 5.89, compared to 4.12 for the 30 min sample. This observation aligns with the earlier stability trends. By the 10th day, the absorbance of the 90 min sample decreased to 4.78, yet remained higher than the initial absorbance (4.12) of the 30 min sample, further confirming the prolonged stabilizing effect of extended ultrasonication.

#### 3.2.2. Surfactant Type and Concentration

**Figure 10:**
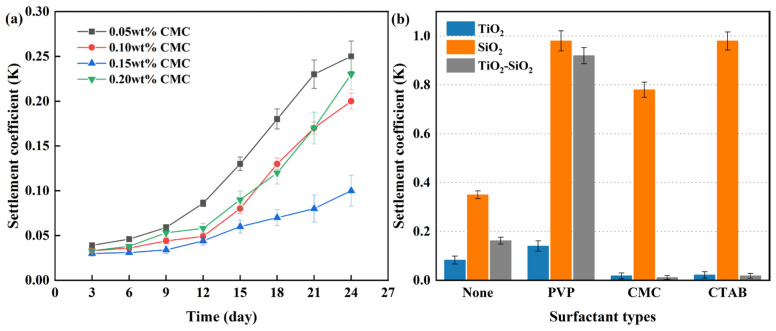
Influence of surfactant concentration (CMC) on the stability of TiO_2_-SiO_2_ nanofluids (**a**), and a comparison of the sedimentation coefficients for different nanofluids after one week under a fixed surfactant concentration of 0.15 wt% CMC with varying surfactant chemistries (**b**).

As shown in Figure 10a, under identical conditions (total particle concentration 0.20 vol%, 1:1 blend of 20 nm TiO_2_ and 50 nm SiO_2_), the CMC surfactant at a concentration of 0.15 wt% demonstrated clearly superior stabilizing performance compared to other concentrations, which aligns with findings reported in earlier studies. For example, Khairul et al. [101] noted in their study of CuO nanofluids that 0.15 wt% SDBS surfactant yielded the best results, exhibiting optimal thermal conductivity and DLS particle size distribution. The sedimentation coefficient was highest for the 0.05 wt% CMC, indicating the poorest stabilization effect, followed by 0.20 wt% CMC.

Figure 10b shows that the anionic surfactant CMC significantly enhanced the stability of both TiO_2_ and TiO_2_-SiO_2_ nanofluids, while the cationic CTAB provided a moderate improvement. This can be attributed to the strong affinity of hydroxyl groups (-OH) present in anionic surfactants toward metal-oxide surfaces. In contrast, the nonionic surfactant PVP promoted sedimentation of the metal-oxide nanoparticles. For the non-metal-oxide SiO_2_, all tested surfactants accelerated particle aggregation and sedimentation under the given conditions.

**Figure 11:**
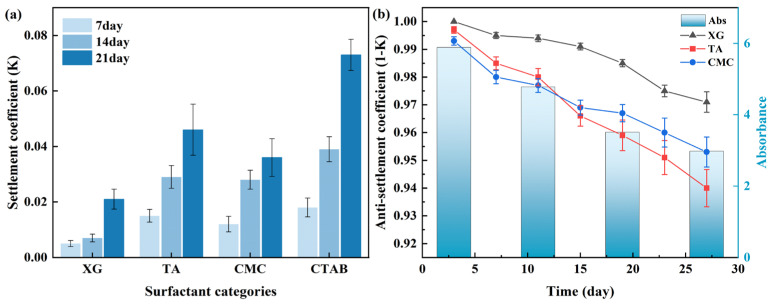
Comparison of sedimentation coefficients for TiO_2_-SiO_2_ nanofluids prepared with different surfactant types at various time points (**a**); correlation between absorbance and sedimentation coefficient for TiO_2_-SiO_2_ (XG) nanofluid (**b**).

Following the determination of optimal parameters for all other influencing factors, the TiO_2_-SiO_2_ hybrid nanofluid was subjected to optimization focused on surfactant non-toxicity and stability. For green surfactants, under identical conditions (0.2 vol% nanoparticle concentration, 90 min ultrasonication, 0.15 wt% surfactant concentration, 20 nm TiO_2_ and 50 nm SiO_2_ particles), the stability of the nanofluid prepared with TA was comparable to that with CMC, as shown in Figure 11a. XG demonstrated even better stabilizing performance. These results reaffirm the superior stabilizing effect of anionic surfactants on metal-oxide nanoparticles and further indicate that not all non-ionic surfactants necessarily induce adverse effects, such as those observed with PVP. The stabilizing effect of XG on nanofluids may be attributed to its ability to reduce the viscosity of the nanofluids, thereby decreasing the sedimentation rate of nanoparticles. The influence of viscosity on stability has also been confirmed in previous studies [102]. Based on quantitative stability analysis via sedimentation coefficient measurements, the following conclusions were drawn: Under the same conditions, the sedimentation coefficient of XG-stabilized nanofluids decreased by 41.67% compared to that of CMC, whereas TA increased it by 27.78%. Thus, XG was determined to be the most effective surfactant.

Finally, a comparative analysis was conducted between sedimentation coefficient and absorbance measurements over a one-month period. For ease of comparison, an inverse sedimentation coefficient (1 − K) was introduced, where lower values correspond to greater sedimentation. As shown in Figure 11b, the observed decrease in the inverse sedimentation coefficient closely matched the reduction in absorbance for the prepared TiO_2_-SiO_2_ nanofluids. The stability of the nanofluids was verified by both characterization methods to decrease with increasing time. This consistency corresponds to the underlying principles shared by both measurement methods, validating the practical utility of the sedimentation coefficient as a stability indicator.

### 3.3. Flow Heat Transfer Experiments

To evaluate the thermal–hydraulic behavior of the nanofluids, experiments were conducted one day after preparation. The Reynolds number (flow rate) was maintained in the range of 350–550 by adjusting the liquid flow rate, with a heating power of 30 W and a constant-temperature tank temperature of 20 °C. Using the previously optimized formulation—0.15 wt% XG, 20 nm TiO_2_, 50 nm SiO_2_, and a 1:1 blending ratio—the performance of TiO_2_, SiO_2_, and TiO_2_-SiO_2_ nanofluids was systematically compared. After each nanofluid test, the MCHS was thoroughly cleaned to eliminate the possibility of sediment formation, thereby enhancing the accuracy of each experimental run.

#### 3.3.1. Thermal Performance

**Figure 12:**
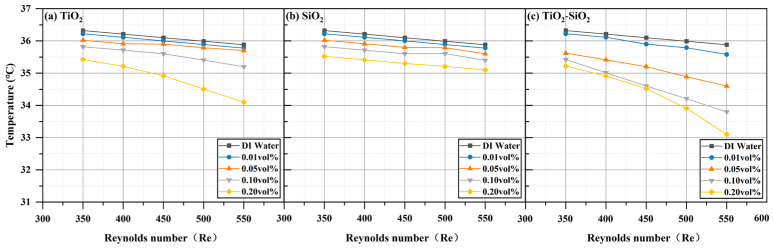
Comparison of wall temperature (*T_w_*) for three nanofluids across different concentrations.

**Figure 13:**
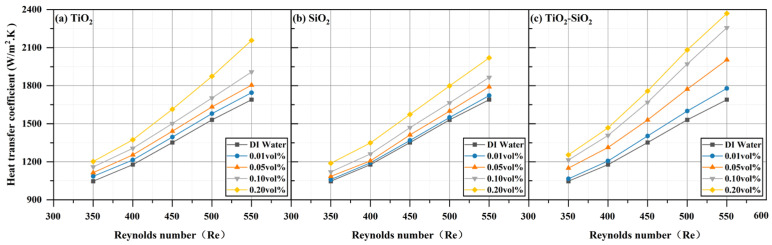
Comparison of convective heat transfer coefficient (*h*) for three nanofluids across different concentrations.

**Figure 14:**
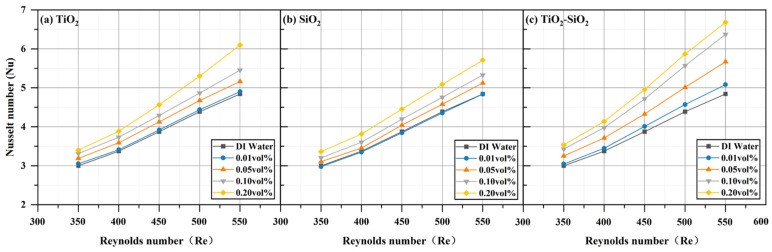
Comparison of Nusselt number (*Nu*) for three nanofluids across different concentrations.

**Figure 15:**
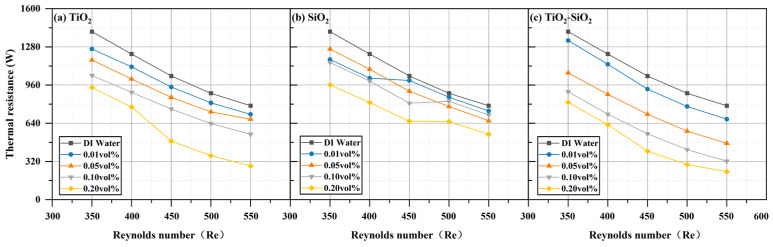
Comparison of thermal resistance (*R_th_*) for three nanofluids across different concentrations.

All three nanofluids (TiO_2_, SiO_2_, and TiO_2_-SiO_2_) at four different concentrations exhibited enhanced heat transfer performance compared to deionized water, with performance increasing as the Reynolds number increased. This phenomenon is also reflected in the research by Emmanuel O. Atofarati et al. [103]. The most pronounced enhancement was observed at a Reynolds number of 550 and a concentration of 0.20 vol%, as shown in Figure 12. The reduction in wall temperature ($T_{W}$) relative to deionized water reached 4.96% for TiO_2_, 2.23% for SiO_2_, and 7.75% for TiO_2_-SiO_2_ nanofluids. For TiO_2_-SiO_2_ nanofluids, the higher the concentration, the more pronounced the effect of the Reynolds number. Figure 13 shows that the convective heat transfer coefficient (*h*) was enhanced by 27.65% for TiO_2_, 19.53% for SiO_2_, and 40.25% for TiO_2_-SiO_2_. It can be observed that as the Reynolds number increases, the heat transfer coefficient rises with the thickening of the thermal boundary layer. Moreover, the addition of nanoparticles to the base fluid significantly enhances the heat transfer coefficient. This is likely attributable to enhanced thermal conductivity, distinct slip phenomena (relative motion between the base fluid and nanoparticles), and surface-scale effects (nanoplate and nanoporous effects) [104,105]. In Figure 14, the Nusselt number (Nu) increased by 25.97% for TiO_2_, 17.95% for SiO_2_, and 37.94% for TiO_2_-SiO_2_. This improvement is mainly attributed to enhanced nanoparticle motion and interparticle collisions, which raise the heat transfer rate as the inlet flow rate increases. Additionally, for TiO_2_-SiO_2_ nanofluids, the Nusselt number gains diminish at concentrations between 0.10 vol% and 0.20 vol%. As presented in Figure 15, thermal resistance ($R_{th}$) decreased by 64.08% for TiO_2_, 30.40% for SiO_2_, and 70.13% for TiO_2_-SiO_2_. Lower thermal resistance indicates better heat dissipation performance of the heat sink, a key characteristic in MCHS applications. This also means that the optimal TiO_2_-SiO_2_ nanofluid prepared in this paper significantly enhances heat dissipation performance. Additionally, it can be observed that the thermal resistance curves of TiO_2_ nanofluids and TiO_2_-SiO_2_ nanofluids are highly similar, with the distinction that the TiO_2_-SiO_2_ nanofluid exhibits more uniform variation with concentration changes.

In summary, the TiO_2_-SiO_2_ hybrid nanofluid showed significantly greater enhancement in heat transfer performance than the single-component systems. This pronounced effect may stem from the previously described mechanism in which smaller TiO_2_ particles fill the gaps between larger SiO_2_ particles, forming efficient “large-particle–small-particle–base-fluid” heat transfer pathways. This structure not only substantially enhances the thermal properties of the working fluid but likely also contributes to the superior colloidal stability observed in the hybrid system.

#### 3.3.2. Hydraulic Performance

**Figure 16:**
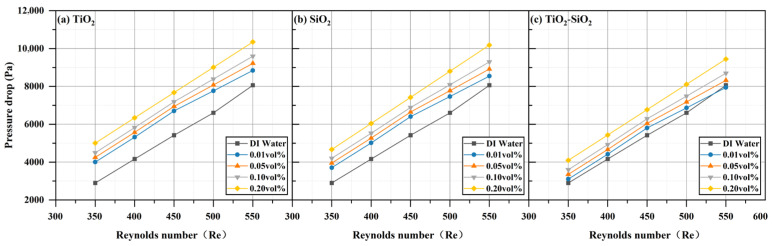
Comparison of pressure drop for three nanofluids across different concentrations.

**Figure 17:**
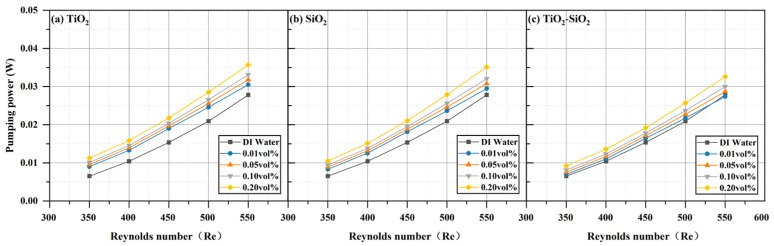
Comparison of pumping power for three nanofluids across different concentrations.

**Figure 18:**
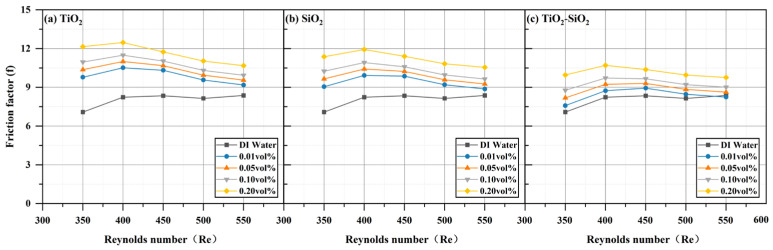
Comparison of friction factor for three nanofluids across different concentrations.

The hydraulic parameters of three nanofluids (TiO_2_, SiO_2_, and TiO_2_-SiO_2_) at four different concentrations all increased compared to those of deionized water and rose further with increasing Reynolds number. This trend aligns with the simulation results reported by Valiyollah Ghazanfari et al. [106] for heat exchangers. The most significant enhancement was observed at a Reynolds number of 550 and a concentration of 0.20 vol%. As shown in Figure 16, the pressure (ΔP) drop increased by 28.36% for TiO_2_, 26.29% for SiO_2_, and 17.18% for TiO_2_-SiO_2_ relative to deionized water. In previous studies, mixed nanofluids often exhibited the undesirable effect of increased pressure drop while enhancing heat transfer [107]. It can be observed that the pressure drop of TiO_2_-SiO_2_ nanofluids exhibits the least variation with concentration. The optimized TiO_2_-SiO_2_ nanofluid in this study exhibits lower pressure drop than single-component nanofluids, indicating that it not only possesses excellent stability and heat transfer performance but also significantly enhances hydraulic performance. The rise in pressure drop can be attributed to the flow velocity: as the inlet flow velocity increases, the boundary layer thickness decreases, thereby increasing shear stress and leading to higher pressure loss. Additionally, for TiO_2_-SiO_2_ nanofluids, the optimization effect on pressure drop exhibits a significant increase in benefit at concentrations ranging from 0.10 vol% to 0.20 vol%. It is noteworthy that even at very low concentrations, both TiO_2_ nanofluids and SiO_2_ nanofluids cause a significant increase in pressure drop. This further demonstrates the substantial hydraulic performance advantages of the optimized TiO_2_-SiO_2_ nanofluid developed in this study. Figure 17 shows that pumping power ($P_{p}$) also increased by 28.36% for TiO_2_, 26.29% for SiO_2_, and 17.18% for TiO_2_-SiO_2_. In nanofluids, the addition of nanoparticles increases both density and viscosity, thereby increasing pumping power requirements. The power required to circulate fluid through the narrow channels of a radiator in a closed loop is referred to as pumping power. Similarly, under identical conditions, the TiO_2_-SiO_2_ nanofluid exhibited the lowest pumping power value, further validating the superior hydraulic performance of this optimized nanofluid. It is noteworthy that, consistent with the previous discussion, even very low concentrations of TiO_2_ nanofluids and SiO_2_ nanofluids result in a significant increase in pumping power. In Figure 18, the friction factor (f) rose by 27.52% for TiO_2_, 25.99% for SiO_2_, and 16.66% for TiO_2_-SiO_2_. As the Reynolds number increased, the friction factor decreased for all three fluids, which is consistent with the laminar flow relationship where the friction factor is inversely proportional to the Reynolds number. Across the entire Reynolds number test range, the friction force of all nanofluids first increased and then decreased, reaching its maximum value at a Reynolds number of 350. Compared to other performance metrics, the variation curve for friction force was more pronounced.

In summary, TiO_2_ nanofluid—a common component in nano-lubricants—exhibited the poorest hydraulic performance, followed by SiO_2_. This may be explained by the inherently lower friction and anti-wear properties of SiO_2_. This characteristic is consistent with the findings of Asif Khan and Muddassir Ali [81] regarding SiO_2_ nanoparticles. The TiO_2_-SiO_2_ hybrid nanofluid, through the previously described “large-particle–small-particle–base-fluid” channel structure, significantly improved lubricating performance, leading to lower friction and reduced pressure drop.

#### 3.3.3. Performance Evaluation Criterion

**Figure 19:**
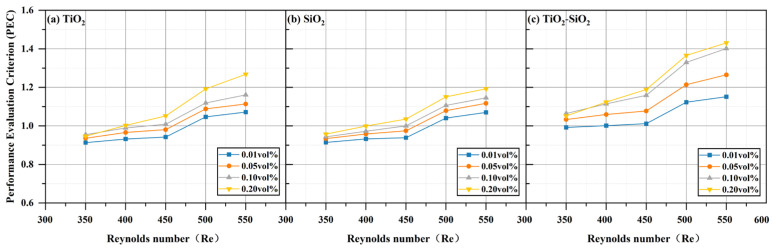
Comparison of performance evaluation criterion (PEC) for three nanofluids across different concentrations.

As shown in Figure 19, the PEC (Performance Evaluation Criterion) increases with increasing Reynolds number. At a Reynolds number of 550, the maximum PEC value for the 0.20 vol% TiO_2_-SiO_2_ nanofluid reaches approximately 1.43. This indicates that hybrid nanofluids in MCHS are more effective than the base fluid (DI water). Additionally, it can be observed that the PEC values of TiO_2_-SiO_2_ nanofluids at 0.10 vol% and 0.20 vol% are quite similar. This implies that a preparation concentration of 0.10 vol% can achieve comparable PEC while reducing costs.

#### 3.3.4. Price Performance Factor

**Figure 20:**
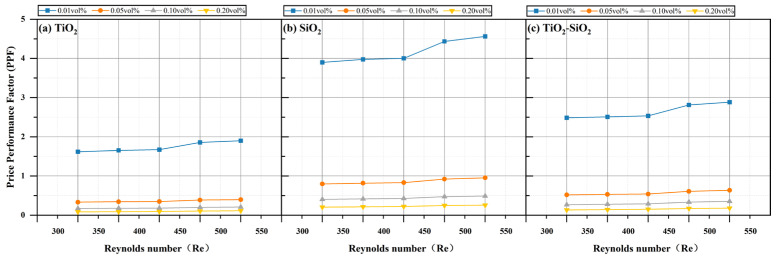
Comparison of price performance factor (PPF) for three nanofluids across different concentrations.

As shown in Figure 20, the PPF also increases with Reynolds number. However, in contrast to the PEC, a lower concentration results in lower cost, while a higher concentration yields lower PPF—implying poorer cost–benefit performance. Under identical conditions, the PPF values of TiO_2_-SiO_2_ nanofluids are consistently higher than those of TiO_2_ nanofluids but lower than those of SiO_2_ nanofluids. Since SiO_2_ nanoparticles require the least mass at the same volume concentration and have the lowest unit price, the SiO_2_ nanofluid achieves the highest PPF cost–benefit factor. This consideration of cost-effectiveness is crucial in large-scale industrial production.

## 4. Conclusions

This study addresses previous challenges and optimizes the preparation parameters of nanofluids. Key findings include:

- The stability of nanofluids does not increase monotonically with decreasing particle concentration. Their stability arises from the combined effects of van der Waals forces, Coulomb forces, and Brownian motion, with an optimal concentration value existing. This study determined the optimal concentrations for TiO_2_, SiO_2_, and TiO_2_-SiO_2_ nanofluids to be 0.10 vol%, 0.10 vol%, and 0.20 vol%, respectively.
- Regarding surfactants, the hydroxyl group (-OH) in anionic types exhibits a strong affinity for metal surfaces. Different nonionic surfactants exert varying effects on nanofluid stability, suggesting that mechanisms beyond electrostatic stabilization and steric stabilization may be involved—such as the influence of fluid viscosity on the sedimentation rate of nanofluid particles.
- A 90 min ultrasonic treatment activates the surfaces of metal-oxide nanoparticles like TiO_2_, significantly enhancing the dispersion stability of the nanofluid while reducing interparticle repulsion forces. This treatment decreases the sedimentation coefficient and markedly improves the results from DLS testing. It prevents agglomeration and sedimentation of the nanofluid for at least 10 days.
- The thermal performance of nanofluids increases with rising particle volume concentration. Under identical conditions, mixed nanofluids exhibit superior thermal properties compared to deionized water. The TiO_2_-SiO_2_ nanofluid boosts the convective heat transfer coefficient by 40.25%, increases the Nusselt number by 37.94%, and achieves a maximum PEC value of 1.43.
- The mixed nanofluid with size-graded particles significantly enhances dispersion stability. At a total particle concentration of 0.20 vol% and a Reynolds number of 550, the sedimentation coefficient of the mixed nanofluid composed of 20 nm TiO_2_ and 50 nm SiO_2_ nanoparticles was only 61.11% of that of a mixed nanofluid with particles of the same size, indicating significantly reduced sedimentation.
- The mixed nanofluid with particles of different sizes significantly enhanced hydraulic performance. At a total particle concentration of 0.20 vol% total particle concentration and a Reynolds number of 550, the hybrid nanofluid composed of 20 nm TiO_2_ and 50 nm SiO_2_ nanoparticles exhibited the smallest pressure drop compared to single-component fluids. This value increased by only 17.18% relative to deionized water, representing 91.29% and 92.79% of the pressure drops generated by TiO_2_ and SiO_2_ nanofluids, respectively
- Absorbance data measured by UV-visible spectroscopy validated the reliability of the visually determined sedimentation coefficient. Furthermore, the study determined that a dilution ratio of 1:30 yields optimal absorbance test results for emulsified TiO_2_-based nanofluids at a concentration of 0.20 vol%.
- As nanoparticle concentration increases, PPF values decrease, leading to reduced cost-effectiveness. At equivalent volume concentrations, SiO_2_ nanoparticles exhibit the highest PPF cost-effectiveness coefficient due to their minimal mass and lowest unit price.

Despite employing diverse experimental variables and stability characterization methods, this study remains constrained by temporal limitations. Factors such as pH have not been experimentally validated, preventing determination of whether surfactants deemed ineffective might exhibit different behavior under adjusted pH conditions. Due to laboratory equipment constraints, testing of emulsified TiO_2_-SiO_2_ nanofluids was confined to heat transfer studies, excluding applications in lubrication, batteries, and photothermal conversion. This study diluted only the supernatant fraction. Although it may be interesting to analyze the sedimentation effect, this can distort the actual particle distribution. This could be combined by periodical measurements of the whole suspension (after being manual shaking) to analyze possible agglomeration effects, which may serve as a suggestion for future investigations. Furthermore, as future datasets expand to encompass broader experimental conditions and variables, advanced machine learning methods—such as artificial neural networks, Gaussian process regression, and support vector machines—can be employed to further assist in designing and optimizing subsequent experiments.

## Figures and Tables

**Table 1:** Specifications for Nanoparticle Selection.

Name	Size (nm)	Density (kg/m^3^)	Specific Heat Capacity (J/Kg⋅k)	Thermal Conductivity (W/m^2^⋅K)	Price (USD/g)
TiO_2_	20	4260	710	10	0.04407
SiO_2_	20	2200	765	1.4	0.03762
SiO_2_	50	2200	765	1.4	0.03554

**Table 2:** Surfactants used in this study.

Names	Abbreviations	Types
Carboxymethyl cellulose	CMC	Anionic
Tannic acid	TA	Anionic
Cetyltrimethylammonium bromide	CTAB	Cationic
Polyvinylpyrrolidone	PVP	Non-ionic
Xanthan gum	XG	Non-ionic

**Table 3:** Zeta Potential Analysis of Different Nanofluids.

Particle Matters	Ultrasound Time	Surfactant Types	Zeta
TiO_2_	90 min	CMC	−5.70 mV
TiO_2_	30 min	CMC	−53.1 mV
TiO_2_-SiO_2_	90 min	CMC	−32.3 mV
TiO_2_-SiO_2_	30 min	CMC	−26.8 mV
TiO_2_-SiO_2_	90 min	XG	−28.8 mV
TiO_2_-SiO_2_	30 min	XG	−0.41 mV

## Data Availability

The original contributions presented in this study are included in the article. Further inquiries can be directed to the corresponding authors.

## Abbreviations

**Acronym**	
Abs	Absorbance
DLS	Dynamic light scattering
CMC	Carboxymethyl cellulose
CTAB	Cetyltrimethylammonium bromide
DW	Deionized water
HTC	Heat transfer coefficient
MCHS	Minichannel heat sink
PEC	Performance evaluation criteria
PVP	Polyvinylpyrrolidone
SiO_2_	Silicon dioxide, silica
TiO_2_	Titanium dioxide, titania
TA	Tannic acid
XG	Xanthan gum
UV-Vis	Ultraviolet–Visible
**Symbols**	
*A*	Cross-sectional area, m^2^
*Aw*	Surface area of the wall m^2^
*Cp*	Heat Capacity, J/kg·K
*dbf*	Molecular diameter of the base fluid, m
*dh*	Hydraulic diameter, m
*f*	Friction factor
*H*	Height, m
*h*	Heat transfer coefficient, W/m^2^·K
*K*	Nanofluid sedimentation rate
*k*	Thermal conductivity, W/m.K
*L*	Length, m
*m*	Mass kg
$\dot{m}$	Mass flow rate, kg/s
*N*	Number
*Nu*	Nusselt Number
*n*	Particle shape factor (empirical)
*Q*	Rate of heat flow, W
*Re*	Reynolds Number
*T*	Temperature, °C
$\dot{V}$	Volumetric flow rate, m^3^/s
*W*	Width, m
**Greek Symbols**	
*μ*	Viscosity, kg/(m·s)
*ρ*	Density, kg/m^3^
*φ*	Particle concentration
*Φ*	Volume concentration, vol%
*ν*	Velocity, m/s
**Subscript**	
*b*	Base
*bf*	Base fluid
*C*	Contraction coefficient
*c*	Coolant
*E*	Expansion coefficient
*eff*	Effective
*fin*	Fin
*hs*	Heat sink
*h*	Hydraulic
*hnf*	Hybrid nanofluid
*in*	Inlet
*m*	Measured
*nf*	Nanofluid
*np*	Nanoparticle
*O*	Reference
*out*	Outlet
*p*	Power
*t*	Total
*w*	Wall
